# Symptom assessment related to the menstrual cycle to predict endometriosis and adenomyosis in university students

**DOI:** 10.5935/1518-0557.20240091

**Published:** 2025

**Authors:** Beatriz Memória Feitosa, Cristiano César Rodrigues Augusto Gonçalves, Beatriz Vieira Cavalcante, André Lucas Grangeiro de Sá Barreto Lima, Caroline Martins de Souza, Larissa Brandão Joventino, Edward Araujo Júnior, Marcelo Borges Cavalcante

**Affiliations:** 1 Medical School, University of Fortaleza (UNIFOR), Fortaleza-CE, Brazil; 2 Department of Obstetrics, Paulista School of Medicine - Federal University of São Paulo (EPM-UNIFESP), São Paulo-SP, Brazil; 3 Discipline of Woman Health, Municipal University of São Caetano do Sul (USCS), São Caetano do Sul-SP, Brazil; 4 Graduate Program in Medical Sciences, University of Fortaleza (UNIFOR), Fortaleza-CE, Brazil; 5 CONCEPTUS - Reproductive Medicine, Fortaleza-CE, Brazil

**Keywords:** endometriosis, adenomyosis, dysmenorrhea, pelvic pain, university students

## Abstract

**Objective:**

To assess the prevalence of self-reported symptoms of endometriosis and adenomyosis among university students and identify potential predictors of these diseases among these symptoms.

**Methods:**

This cross-sectional study was conducted at a private university in northeastern Brazil. The students were asked to complete an electronic questionnaire using a Google Form link. Participants were asked about general information, the menstrual cycle, and bleeding symptoms. The electronic questionnaire results were compared between two groups: students who self-reported endometriosis/adenomyosis (ENDO/ADENO) and students who self-reported no endometriosis/adenomyosis (NO ENDO/ADENO).

**Results:**

Four (2.9%) students self-reported adenomyosis alone, 26 (18.6%) students self-reported endometriosis alone, and two (1.4%) students selfreported an associated diagnosis of endometriosis and adenomyosis. Participants were divided into two groups: ENDO/ADENO (n=32) and NO ENDO/ADENO (n=108).

Participants in the ENDO/ADENO group reported more severe dysmenorrhea, worsening dysmenorrhea in the last 12 months, frequent absence from class, dyspareunia, and dysuria. Worsening dysmenorrhea was a predictor of endometriosis/adenomyosis in university female students (odds ratio = 5.73; 95% confidence interval, 1.91-17.22, *p*=0.002).

**Conclusions:**

The assessment of menstrual cycle symptoms can be used as a screening tool for patients at risk of endometriosis/adenomyosis. The progressive worsening of dysmenorrhea in the last 12 months was a predictor of endometriosis/adenomyosis diagnosis.

## INTRODUCTION

Endometriosis is a gynecological disease characterized by the presence of endometrial tissue (composed of glands and stroma) outside the uterine cavity (Giudice, 2010; Zondervan *et al.*, 2020; Taylor *et al.*, 2021). Endometriosis affects about 10% of adolescents and women of reproductive age worldwide (Giudice, 2010; Fuldeore & Soliman, 2017; Zondervan *et al.*, 2020; Taylor *et al.*, 2021). Endometriosis has traditionally been associated with two main symptoms: pelvic pain (particularly during menstrual bleeding, known as dysmenorrhea) and infertility (Giudice, 2010; Zondervan *et al.*, 2020; Taylor *et al.*, 2021; Ottolina *et al.*, 2024).

Adenomyosis develops when endometrial tissue grows within the uterine wall (myometrium) (Levy *et al.*, 2013; Upson & Missmer, 2020). The true prevalence of adenomyosis is unknown. Adenomyosis was found in histopathological findings of hysterectomies in studies ranging from 8.8% to 61.5% (Upson & Missmer, 2020). Abnormal uterine bleeding, dysmenorrhea, and infertility are common symptoms in patients with adenomyosis (Levy *et al.*, 2013). Interestingly, numerous studies have found a strong association between adenomyosis and endometriosis (Leyendecker *et al.*, 2015).

Endometriosis and adenomyosis negatively impact women’s quality of life. Endometriosis has been associated with an increase in disruptions in the academic, work, and social lives of young women (Gupta *et al.*, 2021; Bell *et al.*, 2023). Consequently, endometriosis causes large economic losses in Australia, with an estimated annual cost of roughly $16,970 to $20,898 per woman per year, with most of the expenses attributable to absence from work (Armour *et al.*, 2019; Bell *et al.*, 2023). The economic burden in the United States is estimated to be $78-$119 billion per year (Simoens *et al.*, 2012; Ellis *et al.*, 2022). Endometriosis has comparable direct and indirect costs with other chronic diseases, such as heart disease and diabetes mellitus (Simoens *et al.*, 2012; Armour *et al.*, 2019).

The diagnosis of endometriosis and adenomyosis remains a major challenge for general practitioners and reproductive medicine specialists. According to studies, the interval between the first clinical manifestations and the diagnosis of endometriosis can take up to 12 years, particularly in young women (Hadfield *et al.*, 1996; Arruda *et al.*, 2003; Husby *et al.*, 2003). The “normalization,” nonspecificity, and multiplicity of endometriosis symptoms, as well as the lack of specific biomarkers and variation in the intensity of symptoms, make endometriosis and adenomyosis clinical diagnosis challenging (Zondervan *et al.*, 2020). Dysmenorrhea (62.2%), chronic pelvic pain (56.8%), deep dyspareunia (54.7%), cyclical intestinal complaints (48.3%), infertility (39.8%), and cyclical urinary complaints (11.7%) were the most commonly reported symptoms among a group of Brazilian women who underwent laparoscopy with histological confirmation of endometriosis (Bellelis *et al.*, 2010).

The worsening of quality of life, increased risk of mental disorders, disease progression with the intensification of symptoms, development of central sensitization of pelvic pain, and erosion of the physician-patient relationship are some of the consequences of endometriosis diagnosis and treatment delays (Zondervan *et al.*, 2020). Furthermore, the absence of adequate clinical-surgical management and reproductive planning for women with undiagnosed endometriosis can have serious reproductive consequences, including accelerated loss of ovarian reserve, infertility, and the need for assisted reproductive technology to achieve pregnancy (Tanbo & Fedorcsak, 2017; Bonavina & Taylor, 2022).

The visualization (often by laparoscopy) of endometriotic lesions, whether confirmed by histology or not, remains the gold standard for diagnosing endometriosis. The use of imaging tests (ultrasound or magnetic resonance imaging) is also recommended for nonsurgical assessment of suspected endometriosis cases (Becker *et al.*, 2022). Ideally, patients suspected of having endometriosis should be referred for imaging or surgical evaluation (Becker *et al.*, 2022; Ottolina *et al.*, 2024). Self-reporting tools based on patient complaints have been proposed to identify women with suspected endometriosis symptoms to reduce diagnostic delay and support early endometriosis treatment (Surrey *et al.*, 2017; Fauconnier *et al.*, 2021; Chapron *et al.*, 2022).

This study aims to assess the prevalence of self-reported symptoms of endometriosis and adenomyosis among Brazilian university students and identify potential predictors of these diseases.

## MATERIAL AND METHODS

This is a cross-sectional study conducted between January and October 2023. The study population consisted of Brazilian female students from the University of Fortaleza (UNIFOR), a private university in Fortaleza, Ceara, in the northeast of Brazil. This study was reported in accordance with The Strengthening the Reporting of Observational Studies in Epidemiology (STROBE) Statement. Non-Brazilians, those under the age of 18 years, those unable to communicate in Portuguese, and those who refused to participate in the study were excluded. Eligible participants were asked to participate in the study using an internal communication platform and social networking application (WhatsApp Messenger). Those who wanted to participate used a Google Form link to access the online questionnaire. An online questionnaire link was accessible for completion between August 1 and September 30, 2023.

### Instrument

The electronic instrument was developed using the Menstrual Disorder of Teenagers Questionnaire (Parker *et al.*, 2010). A pilot study was conducted to test the validity and reliability of the questionnaire. After that, it was revised based on participant feedback and test-retest results. The final questionnaire comprised 27 items divided into three sections: general information, menstrual cycles, and symptoms related to the period (Table 1).

**Table 1:** English version of the questionnaire used in the study.

**Section 1: General Information (9 items)** 1. How old are you? 2. What is your weight? 3. What is your height? 4. How old were you when you got your first period? 5. What course are you enrolled in? 6. How many years have you been in university? 7. Do you have a confirmed diagnosis of any of the following diseases? (endometriosis, adenomyosis, uterine fibroid, polycystic ovary syndrome, endometrial polyp, and other diseases)? 8. Do you use any contraceptive method? 9. Which one? (combined pill, hormonal intrauterine device, copper/silver intrauterine device, combined injectable, progesterone injection, male condom, female condom, contraceptive implant, minipill, or other methods)
**Section 2: Menstrual cycles (17 items)** 10. Do you have periods? 11. Over the past 12 months, have your periods been (regular, irregular, or don't know). Regular = the time between periods is typically about the same length, irregular = if the length of time between periods often changes. 12. What is the usual number of days from the first day of bleeding at one period to the first day of bleeding at your next period? 13. Please tick the column that indicates the heaviness of your bleeding for each day of your period (how it usually is: light, medium, or heavy). 14. Does your bleeding contain clots (sometimes, most of the time, all the time, or not)? 15. Do you miss class because of your periods (no; yes, every month; yes, more than half the months of the year; yes, less than half the months of the year)? 16. If you answered YES to the previous question, why are you missing class (menstrual cramps, excessive bleeding, nausea/vomiting, other reasons)? 17. Please rate any period pain you have had over the past 12 months (VAS). 18. How do you describe this pain (generalized pain, cramping pain, stinging pain, other type)? 19. If you have period pain, do you take medication (I don't take medication; yes, medication at home; yes, medication in a health unit)? 20. Has your period pain worsened over the past 12 months? 21. Does the pain spread to the back, in the lower back? 22. Does the pain spread to the legs and hips? 23. Does the pain become disabling for daily activities? 24. Does the pain prevent you from doing any physical activity? 25. Do you have strong, sharp, and deep internal pain during sexual intercourse? 26. Do you experience pain in certain positions during sexual intercourse?
**Section 3: Symptoms related to the period (1 item)** 27. In the last 12 months, have you experienced any of the following symptoms related to your period: nausea/vomiting, bloating (swollen tummy), diarrhea, constipation, heartburn/reflux, changes in appetite, aching down the legs, pelvic pain, lower back pain, pain when urinating, pain when full bladder, blood in urine, urgency for urinate, pain when evacuating, blood in feces, discharge vaginal, irritability, anxiety, and depression?

The electronic questionnaire was developed based on the Menstrual Disorder of Teenagers (MDOT). VAS: visual analog scale.

### General information (nine items)

Participants were asked to provide their age, weight, height, age at first period, course enrolled, number of years at university, history of gynecological diseases (endometriosis, adenomyosis, uterine fibroid, polycystic ovary syndrome, endometrial polyp, and other diseases), and use of a contraceptive method (combined pill, hormonal intrauterine device, copper/silver intrauterine device, combined injectable, progesterone injection, male condom, female condom, contraceptive implant, minipill, or other methods).

### Menstrual cycles (17 items)

The occurrence of periods and the characteristics of menstrual cycles were assessed with the questions listed in Table 1 (section 2). The period pattern (regularity, duration, and intensity of flow), the presence of dysmenorrhea (intensity, pattern, progression, and use of medication), interference with sexual practice, and daily activities, including missing classes, were asked of participants.

### Symptoms related to the period (one item)

Participants were asked about the occurrence of the following symptoms associated with the period: nausea, vomiting, bloating (swollen tummy), diarrhea, constipation, heartburn/reflux, changes in appetite, aching down the legs, pelvic pain, lower back pain, pain when urinating, pain when full bladder, blood in urine, urgency to urinate, pain when evacuating, blood in feces, discharge vaginal, irritability, anxiety, and depression.

### Data analyses and ethics approval

Data were input into an Excel 2020 spreadsheet (Microsoft Corp., Redmond, Washington), and descriptive statistics for survey responses were deduced. Participants were divided into two groups based on whether they self-reported having endometriosis and/or adenomyosis, known as ENDO/ADENO and NO ENDO/ADENO. The variables extracted from the electronic instrument were compared between the two groups (ENDO/ADENO *vs*. NO ENDO/ADENO). Continuous variables were compared using unpaired Student’s *t* test, and categorical variables were compared using the Fisher’s exact test or χ^2^ test in the SPSS™ (SPSS Statistical Software V.22.0, IBM Corp., Armonk, New York). Adjusted multivariate analysis was performed using binary logistic regression and included the following independent variables: visual analog scale (VAS) dysmenorrhea (>8), worsening of dysmenorrhea, missing class during the period, dyspareunia, and dysuria. *p* values of less than 0.05 were considered statistically significant. Informed consent was included on the welcome page of the survey. The study was approved by the Research Ethics Committee of the UNIFOR (CAAE: 69273423.6.0000.5052).

## RESULTS

One hundred and forty women agreed to participate in the study. Four (2.9%) students self-reported adenomyosis alone, 26 (18.6%) students self-reported endometriosis alone, and two (1.4%) students self-reported an associated diagnosis of endometriosis and adenomyosis. Thus, study participants were divided into two groups: ENDO/ADENO (32 students) and NO-ENDO/ADENO (108 students).

The demographic characteristics of the study participants are shown in Table 2. The mean age of the participants was 22.4±5.2yr (ranging from 18 to 48yr), and there was no difference between groups (*p*=0.069). The means of weight, height, and body mass index were 62.5±11.8kg, 1.61±0.1m, and 23.9±4.3, respectively. Sixty-seven percent (n=94/140) of participants were normal weight, 2.1% (n=3/140) were underweight, 22.1% (n=31/140) were overweight, and 8.6% (n=12/140) were obese. Anthropometric markers were similar between the two groups.

**Table 2:** Demographic characteristics of participants.

Variable	Total (n=140)	ENDO/ADENO (n=32)	NO ENDO/ ADENO (n=108)	p
Age (years), mean±SD	22.4±5.2	24.3±7.1	21.8±4.4	0.069
Weight (kilogram), mean±SD	62.5±11.8	61.8±11.2	62.7±11.9	0.698
Height (meter), mean±SD	1.61±0.1	1.62±0.1	1.61±0.1	0.785
BMI, mean±SD	23.9±4.3	23.7±4.6	24.1±4.2	0.689
Underweight, n (%)	3 (2.1)	2 (6.3)	1 (0.9)	0.068
Normal weight, n (%)	94 (67.1)	19 (59.4)	75 (69.4)	0.286
Overweight, n (%)	31 (22.1)	10 (31.3)	21 (19.4)	0.157
Obesity, n (%)	12 (8.6)	1 (3.1)	11 (10.2)	0.210
Age at first period (years), mean±SD	11.8±1.4	12.0±1.5	11.7±1.3	0.252
Contraception, n (%)	82 (58.6)	22 (68.8)	60 (55.6)	0.183
Hormonal contraception, n (%)	66 (47.1)	20 (62.5)	46 (42.6)	0.047
Medical student, n (%)	33 (23.6)	8 (25)	25 (23.1)	0.828
Years in university, (years) mean±SD	2.2±1.3	2.3±1.3	2.2±1.3	0.718

ENDO/ADENO: patients who self-reported having a diagnosis of endometriosis and/or adenomyosis. No ENDO/ADENO: patients who self-reported not having a diagnosis of endometriosis and/or adenomyosis. BMI: body mass index. Underweight: BMI under 18.5 kg/m^2^. Normal weight: BMI from 18.5 kg/m^2^ to 24.9 kg/m^2^. Overweight: BMI from 25 kg/m^2^ to 29.9 kg/m^2^. Obesity: BMI 30 kg/m^2^ or more.

The mean age at menarche of the participants was 11.8±1.4 yr (ranging from 9 to 17yr), with no difference between groups (*p*=0.252). The use of contraceptive methods was self-reported by 58.6% (n=82/140) of participants and was comparable between groups (*p*=0.183). The mean of years spent at university one course was 2.2±1.3yr, with no difference between groups (*p*=0.718). A quarter of the women in both groups and the total number of participants were medical students (Table 2).

The frequency of gynecological diseases and menstrual cycle characteristics of the participants were summarized in Table 3. Students in the ENDO/ADENO group self-reported a lower presence of menstrual bleeding (called period), 65.6% (*n*=21/32) *versus* 91.7% (n=99/108), respectively (*p*<0.001). However, the regularity of the period was comparable between groups (*p*=0.052). There was no difference between the duration and intensity (heavy menstrual bleeding and menstrual bleeding with clots) of the period between the groups.

**Table 3:** Frequency of gynecological diseases and characteristics of menstrual cycle.

Variable	Total (n=140)	ENDO/ADENO (n=32)	NO ENDO/ ADENO (n=108)	p
Gynecological disease, n (%)	64 (45.7)	32 (100)	32 (29.6)	< 0.001
Adenomyosis, n (%)	4 (2.9)	4 (12.5)	0 (0)	0.002
Endometriosis, n (%)	28 (20)	28 (87.5)	0 (0)	< 0.001
Endometriosis and adenomyosis, n (%)	2 (1.4)	2 (1.4)	0 (0)	0.498
Mullerian anomalies, n (%)	1 (0.7)	0 (0)	1 (0.9)	1
PCOS, n (%)	30 (21.4)	0 (0)	30 (27.8)	< 0.001
Abnormal uterine bleeding, n (%)	1 (0.7)	0 (0)	1 (0.9)	1
Have period, n (%)	120 (85.7)	21 (65.6)	99 (91.7)	< 0.001
Regular period, n (%)	63 (52.5)	7 (33%)	56 (56.6)	0.052
Duration of regular cycles, (days), mean±SD	27.6±2.8	27.6±2.8	27.6±2.7	0.846
Heavy menstrual bleeding on day 1, n (%)	46 (32.9)	13 (40.6)	33 (30.6)	0.286
Heavy menstrual bleeding on day 2, n (%)	81 (57.9)	21 (65.6)	60 (55.6)	0.310
Heavy menstrual bleeding on day 3, n (%)	54 (38.6)	14 (43.8)	40 (37)	0.493
Menstrual bleeding with clots, n (%)	116 (82.9)	26 (81.2)	90 (83.3)	0.783
VAS dysmenorrhea (grade), mean±SD	6.1±2.4	7.5±2.5	5.7±2.2	< 0.001
VAS dysmenorrhea (≥ 8), n (%)	41 (29.3)	20 (62.5)	21 (19.4)	< 0.001
Dysmenorrhea is getting worse, n (%)	61 (43.6)	26 (81.3)	35 (32.4)	< 0.001
Take medication for pain, n (%)	104 (74.3)	24 (75)	80 (74.1)	0.916
Missing class during period, n (%)	44 (31.4)	17 (53.1)	27 (25)	< 0.001

ENDO/ADENO: patients who self-reported having a diagnosis of endometriosis and/or adenomyosis. No ENDO/ADENO: patients who self-reported not having a diagnosis of endometriosis and/or adenomyosis. PCOS: polycystic ovary syndrome. Regular period Variable= (number of patients with regular periods / number of patients who have periods) x 100. Bleeding with clots: clots present in most or all periods. VAS: Visual Analogue Scale measures pain intensity. It has two end points representing 0 (‘no pain’) and 10 (‘pain as bad as it could possibly be’).

The mean of the visual analog scale (VAS) dysmenorrhea was higher in the ENDO/ADENO group (7.5±2.5 *vs*. 5.7±2.2, *p*<0.001). Participants in the ENDO/ADENO group also had a higher percentage of severe dysmenorrhea (VAS≥8) (62.5% [n=20/32] *vs*. 19.4% [n=21/108], *p*<0.001). Dysmenorrhea worsening over the last 12 months was significantly more common among students in the ENDO/ADENO group than the NO ENDO/ ADENO group (81.3% [n=26/32] *vs*. 32.4% [n=35/108], *p*<0.001). However, there was no difference in the need for medicine to control dysmenorrhea between groups. Participants in the ENDO/ADENO group reported missing classes more frequently during the periods (53.1% [n=17/32] *vs*. 25% [n=27/108], *p*<0.001).

The frequency of symptoms self-reported by study participants is presented in Table 4. The occurrence of most symptoms was comparable between the two groups. Only dyspareunia (50% [n=16/32] *vs*. 17.6% [*n*=19/108], *p*<0.001) and dysuria (31.25 [n=10/32] *vs*. 11.1% [n=12/108], *p*=0.005) were significantly more common among students in the ENDO/ADENO group.

**Table 4:** Frequency of gynecological diseases and characteristics of menstrual cycle.

Variable	Total (n=140)	ENDO/ADENO (n=32)	NO ENDO/ADENO (n=108)	p
Dyspareunia, n (%)	35 (25)	16 (50)	19 (17.6)	< 0.001
Nausea/Vomiting, n (%)	65 (46.4)	19 (59.4)	46 (42.6)	0.094
Bloating (swollen tummy), n (%)	128 (91.4)	29 (90.6)	99 (91.7)	0.853
Diarrhoea, n (%)	93 (66.4)	20 (62.5)	73 (67.6)	0.592
Constipation, n (%)	81 (57.8)	21 (65.6)	60 (55.5)	0.310
Heartburn, n (%)	53 (37.8)	16 (50)	37 (34.3)	0.106
Changes in appetite, n (%)	99 (70.7)	25 (78.1)	74 (68.5)	0.294
Leg pain, n (%)	83 (59.3)	22 (68.7)	61 (56.5)	0.214
Lower back pain, n (%)	104 (74.3)	25 (78.1)	79 (73.1)	0.571
Dysuria, n (%)	22 (17.7)	10 (31.2)	12 (11.1)	0.005
Hematuria, n (%)	18 (12.8)	7 (21.9)	11 (10.2)	0.082
Irritability, n (%)	125 (89.3)	29 (90.6)	96 (88.9)	0.780
Anxiety, n (%)	112 (80)	28 (87.5)	84 (77.8)	0.227
Depression, n (%)	70 (50)	12 (37.5)	58 (53.7)	0.107

ENDO/ADENO: patients who self-reported having a diagnosis of endometriosis and/or adenomyosis. No ENDO/ADENO: patients who self-reported not having a diagnosis of endometriosis and/or adenomyosis.

Binary logistic regression was performed to verify whether missing classes, severe dysmenorrhea, worsening in the last 12 months, dyspareunia, and dysuria are predictive symptoms of endometriosis (Table 5). The statistical model that include dysmenorrhea worsening over the previous 12 months was significant (*χ*^2^[1]=34.7; *p*<0.001, *R*^2^ Negelkerke=0.334). Dysmenorrhea worsening was the only predictor of endometriosis in university female students (odds ratio=5.73; 95% confidence interval, 1.91- 17.22, *p*=0.002; Table 5).

**Table 5:** Binary logistic regression-based on symptoms self-reported by university students.

Variable	Adjusted OR (95% CI)	p
VAS dysmenorrhea (≥ 8)	3.294 (0.769 - 14.106)	0.108
Dysmenorrhea is getting worse	5.731 (1.907 - 17.219)	0.002
Missing class during period	1.057 (0.363 - 3.080)	0.919
Dyspareunia	1.260 (0.275 - 5.784)	0.766
Dysuria	1.416 (0.425 - 4.718)	0.571

OR: odds ratio; CI: confidence interval; VAS: visual analogue scale. VAS: Visual Analogue Scale measures pain intensity. It has two end points representing 0 (‘no pain’) and 10 (‘pain as bad as it could possibly be’).

## DISCUSSION

According to studies, the prevalence of endometriosis remains poorly known, ranging from 2% to 10% in the general population, but can reach 50% in infertile women (Eskenazi & Warner, 1997; Meuleman *et al.*, 2009; Zondervan *et al.*, 2020). In addition, the prevalence of endometriosis in different age groups and based on the intensity of symptoms, particularly pelvic pain, remains unknown. Our study found a 21.4% prevalence of endometriosis associated or not with adenomyosis, which is higher than the general population prevalence but comparable with earlier studies with young women or those who self-reported severe dysmenorrhea (Ragab *et al.*, 2015; Zannoni *et al.*, 2024). In our sample, the prevalence of adenomyosis associated or not with endometriosis (4.2%, 6/140) was low compared with previous studies, which ranged from 1% to 70% (Struble *et al.*, 2016; Zannoni *et al.*, 2024; Exacoustos *et al.*, 2022). This discrepancy in adenomyosis prevalence reported in the literature is attributable to different diagnostic criteria, different patient populations, differences in tissue sample sizes, and possible bias in histopathological analysis (Struble *et al.*, 2016).

Endometriosis is a risk factor for women who complain (cyclical and noncyclical) of dysmenorrhea, deep dyspareunia, dysuria, dyschezia, painful rectal bleeding or hematuria, shoulder tip pain, catamenial pneumothorax, cyclical cough/hemoptysis/chest pain, cyclical scar swelling and pain, fatigue, and infertility (Becker *et al.*, 2022; Mitchell *et al.*, 2024). Dysmenorrhea is a common complaint among young women, affecting up to 90% of adolescents (Klein & Litt, 1981; Andersch & Milsom, 1982; Hillen *et al.*, 1999; Banikarim *et al.*, 2000; Martire *et al.*, 2023; Oliveira *et al.*, 2024). However, studies suggest that around 14%-23% of adolescents suffer from severe dysmenorrhea (Klein & Litt, 1981; Fisher *et al.*, 1989; Teperi & Rimpelä, 1989; Wilson & Keye, 1989; Martire *et al.*, 2023). Studies show that about 47%-73% of adolescents with severe dysmenorrhea are diagnosed with endometriosis (Bullock *et al.*, 1974; Reese *et al.*, 1996; Martire *et al.*, 2023). Thus, the results in our study are consistent with previous studies. Several studies have already described the relationship between the presence and severity of dysmenorrhea and the occurrence of endometriosis (Porpora *et al.*, 1999; Calhaz-Jorge *et al.*, 2004; Van Niekerk *et al.*, 2022; El-Hadad *et al.*, 2023).

Porpora *et al.* (1999) observed a correlation between the total pain score and deep endometriosis on the uterosacral ligaments, peritoneal adhesions, and extent of adnexal adhesions. The authors proposed that the presence and severity of dysmenorrhea are predictors of endometriosis (Porpora *et al.*, 1999). Recently, El-Hadad *et al.* (2023) also noted that dysmenorrhea is a predictor of endometriosis, particularly with onset >3 yr after menarche. Other studies have also suggested that dysmenorrhea may be a predictor of endometriosis/adenomyosis (Peterson *et al.*, 2013; Heitmann *et al.*, 2014; Ashrafi *et al.*, 2016; Fuldeore & Soliman, 2017; Saha *et al.*, 2017). In our study, dysmenorrhea was more severe in the endometriosis/adenomyosis group; however, dysmenorrhea was not considered a good predictor after multivariate analysis. Progressive dysmenorrhea is a clinical manifestation common to endometriosis and adenomyosis (Struble *et al.*, 2016; Zondervan *et al.*, 2020; Becker *et al.*, 2022). It occurs when the intensity and duration of pain during periods increases over time. Few studies found that increased dysmenorrhea severity was a good predictor of endometriosis (Forman *et al.*, 1993; Eskenazi *et al.*, 2001; Hsu *et al.*, 2010). In our study, dysmenorrhea worsening in the last 12 months was a good predictor of endometriosis.

Other predictive symptoms for endometriosis have also been studied (Heitmann *et al.*, 2014; Ashrafi *et al.*, 2016; Kayani *et al.*, 2016; Fuldeore & Soliman, 2017; Van Niekerk *et al.*, 2022). In our study, dyspareunia and dysuria were more common in students who self-reported having endometriosis and/or adenomyosis. However, multivariate analysis indicates that this complaint does not predict endometriosis/adenomyosis. Although dyspareunia and dysuria are more common among women with endometriosis, they are not specific symptoms of endometriosis/ adenomyosis (Agarwal *et al.*, 2019; Becker *et al.*, 2022).

Endometriosis/adenomyosis is often the cause of life disruptions (Zondervan *et al.*, 2020; Gupta *et al.*, 2021; Becker *et al.*, 2022). Gupta *et al.* (2021) found that undergraduate students with endometriosis symptoms have a high level of life disruption (88% any disruption, 82.7% social, 58.8% academic, and 34.4% work). A large Australian study of over 4,000 young women aged 13-25yr found that more than a third of students had missed at least one class due to menstrual symptoms in the last three menstrual cycles. In addition to the loss of academic activities, around 70% of students reported difficulties concentrating during menstrual bleeding (Armour *et al.*, 2020). In our study, students with endometriosis/adenomyosis missed twice as many classes as students who did not self-report an endometriosis/adenomyosis diagnosis.

### Strength and limitations

This study has several strengths. First, it identified the prevalence of endometriosis/adenomyosis in university students in northeastern Brazil, a population that has not yet been studied. Second, it identified the pattern of endometriosis/adenomyosis symptoms in young women, contributing to a better understanding of this gynecological disease. Third, the study found that worsening dysmenorrhea may be a predictor of endometriosis/adenomyosis in young women. Consequently, our results may contribute to reducing delays in diagnosing endometriosis/adenomyosis and reducing complications in the medium and long term.

One important limitation is the study design, which was a cross-sectional study with questionnaires completed individually by participants. Another limitation is the absence of radiologic studies or surgery to confirm the participant’s self-reported diagnosis. However, the questionnaire was based on a previously validated instrument in the literature. In our cross-sectional study, one limitation is that the number of participants was slightly below the recommended sample size calculated to ensure sufficient statistical power and representativeness. This limitation may reduce the precision of our estimates. With a smaller sample size, the variability within the data is less likely to represent the diversity present in the entire population, thus increasing the margin of error and potentially leading to biased results. Additionally, we highlight the need for external validation of our results. External validation is crucial to confirm that the observed associations are not unique to our sample and can be generalized to broader populations.

## CONCLUSION

The assessment of symptoms related to the menstrual cycle can be used as a screening tool for patients at risk of endometriosis/adenomyosis. The progressive worsening of dysmenorrhea in the last 12 months was a predictor of endometriosis/adenomyosis diagnosis.
